# Coat’s like vasculopathy in leber congenital amaurosis secondary to homozygous mutations in *CRB1*: a case report and discussion of the management options

**DOI:** 10.1186/s13104-016-1917-6

**Published:** 2016-02-13

**Authors:** Somar M. Hasan, Arwa Azmeh, Osama Mostafa, Andre Megarbane

**Affiliations:** Department of Ophthalmology, Damascus University, Almouassat University Hospital, Damascus, Syria; Al-Jawhara Center, Arabian Gulf University, Manama, Kingdom of Bahrain​

**Keywords:** Leber congenital amaurosis, Coat’s like vasculopathy, Retinitis pigmentosa, *CRB1*

## Abstract

**Background:**

Mutations in the *CRB1* gene are associated with variable phenotypes of severe retinal dystrophies, and retinal dystrophies resulting from *CRB1* mutations may be accompanied by specific fundus features such as coat’s like vasculopathy in retinitis pigmentosa patients. This is the first report of the occurrence of coat’s like vasculopathy in a patient diagnosed with Leber congenital amaurosis caused by a *CRB1* mutation.

**Case presentation:**

An 18-year old Syrian female patient presented with bilateral gradual loss of vision since early childhood, with recent deterioration in her left eye. She appeared to have an asymmetric bilateral coat’s like vasculopathy which was more severe in the left eye. The diagnosis of Leber congenital amaurosis was suggested, and a genetic *CRB1* sequencing for the patient and her two younger siblings, who also had severe vision loss, was done, upon which the diagnosis of Leber congenital amaurosis associated with exudative retinal detachment due to coat’s like vasculopathy was made. Treatment with panretinal photocoagulation was attempted in the worse left eye, but with no improvement. As the disease suddenly progressed in both eyes, pars plana vitrectomy with endolaser and silicone oil tamponade was performed in the better right eye which led to anatomical stabilization of the case without improvement in the visual acuity.

**Conclusion:**

Leber congenital amaurosis is reported to be associated with multiple systemic and ocular findings, none of which is coat’s like vasculopathy. *CRB1* gene mutations are associated with remarkable retinal findings in patients with retinitis pigmentosa and other fundus dystrophies. In this unique case we are reporting the incidence of coat’s like vasculopathy in a patient diagnosed with Leber congenital amaurosis caused by *CRB1* gene mutation, and its management. *CRB1* mutant patients should be followed up closely as sudden progression can have permanent poor outcomes and as early management is vital in such cases.

## Background

Originally described by Theodore Leber in 1869 [1], Leber congenital amaurosis (LCA; MIM# 204000) is the earliest and most severe form of all hereditary retinal dystrophies, responsible for congenital blindness [1]. LCA accounts for at least 5 % of all retinal dystrophies and is one of the main causes of blindness in children [2].

The clinical features of LCA patients usually include roving eye movements/nystagmus, digito-ocular signs (eye poking or rubbing), an apparently normal or salt-and-pepper pigmented fundus and severely reduced or absent scotopic and photopic electroretinogram (ERG), Although it is an early-onset and severe disease, LCA has a variable expression, which may reflect, at least in part, its high genetic heterogeneity [3].

Mutations in the *CRB1* gene are one of the at least 17 known gene mutations that are accused to cause LCA, and *CRB1* mutations are associated with variable phenotypes of severe retinal dystrophies, ranging from LCA to rod-cone dystrophy [also called retinitis pigmentosa (RP)]. Moreover, RP resulting from *CRB1* mutations may be accompanied by specific fundus features: preservation of the para-arteriolar retinal pigment epithelium (PPRPE) and retinal telangiectasia with exudation (also referred to as coats-like vasculopathy) [4], which is a well-known, although rare, complication of RP [5].

In severe cases this disorder may progress to total retinal detachment and visual loss in the context of longstanding RP [5]. *CRB1* mutations were reported in a significant proportion of patients with coat’s like RP as high as 55 % [6].

In this case, we report the (first) occurrence of coat’s like vasculopathy in a patient diagnosed with LCA caused by a *CRB1* mutation.

## Case presentation

**Fig. 1:**
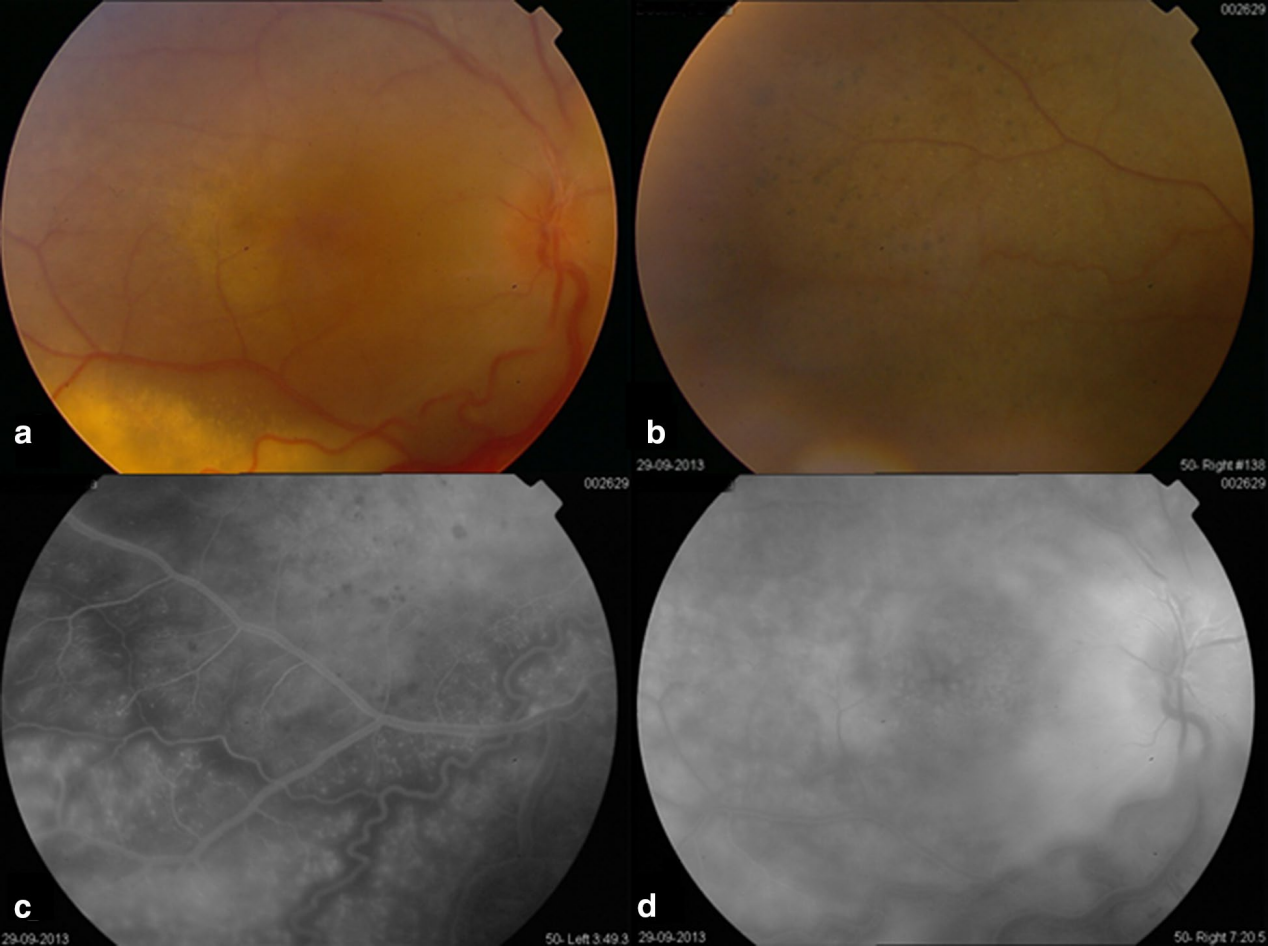
OD Fundus photo and FA of the 18 year old female patient. **a** Abnormal foveal reflex, **b** Peripheral nummular pigmentations in the superior attached retina and infero-temporal exudative retinal detachment, with severe telangiectasia and very dense hard exudates. **c**, **d** FA of the same eye

An 18 year old Syrian female, daughter of first-cousins parents, presented with recent deterioration of visual acuity in her left eye, she reported a history of decreased vision bilaterally that started many (undefined) years ago. Visual acuity was (OD: 0.16) and (OS: Perception of light), examination revealed marked relative afferent pupillary defect (RAPD) in the left eye, cycloplegic refraction was OD: +8.00(−1.50X70) and OS: +10.50(−1.25X15), Axial length measurements were: OD: 18.80 mm and OS: 19.10 mm. Cornea and anterior segment examination was within normal limits and dilated fundus examination revealed:

OD: abnormal foveal reflex (Fig. 1a), peripheral nummular pigmentations in the superior attached retina (Fig. 1b) and infero-temporal exudative retinal detachment not threatening the macula, with severe telangiectasia and very dense hard exudates all over the inferior detached retina. Fluorescein angiography (FA) (Fig. 1c, d) revealed areas of capillary non-perfusion with diffuse capillary and focal leakage in the areas of abnormal vessels with extensive leakage in late phases.

**Fig. 2:**
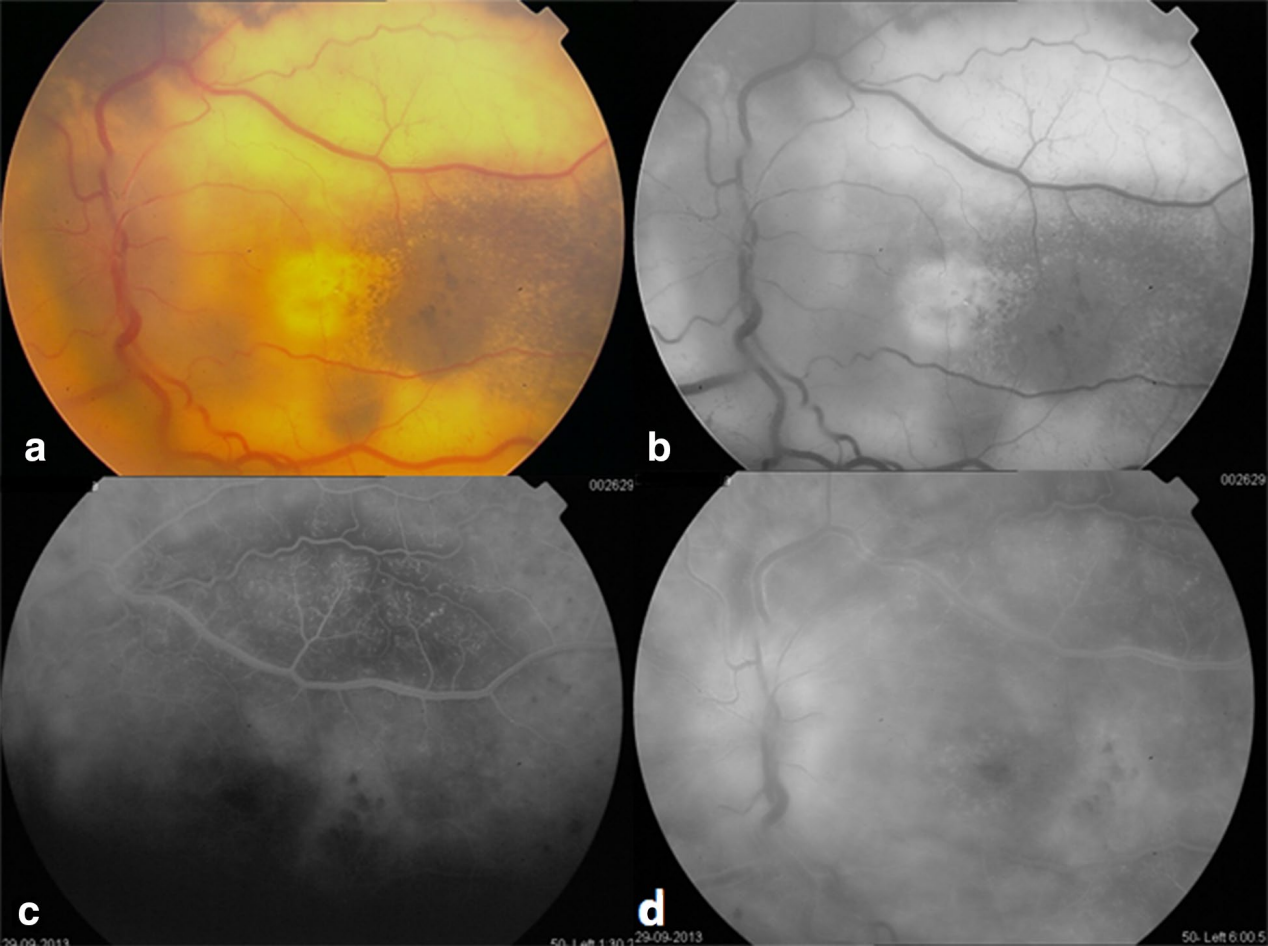
OS Fundus photo and fluorescein angiography of the 18 year old female patient. **a** Mildly elevated subtotal exudative retinal detachment with extensive hard exudates and telangiectatic vessels all over the detached retina, with the same pigmented patches noted in the *right eye*. **b** Red free photo of the same eye. **c**, **d** FA revealing extensive leakage beginning from the early phases and continuing during the whole study time

OS: mildly elevated subtotal exudative retinal detachment with extensive hard exudates and telangiectatic vessels all over the detached retina, with the same pigmented patches noted in the right eye (Fig. 2a, b). FA revealed extensive leakage beginning from the early phases and continuing during the whole study time (Fig. 2c, d).

**Fig. 3:**
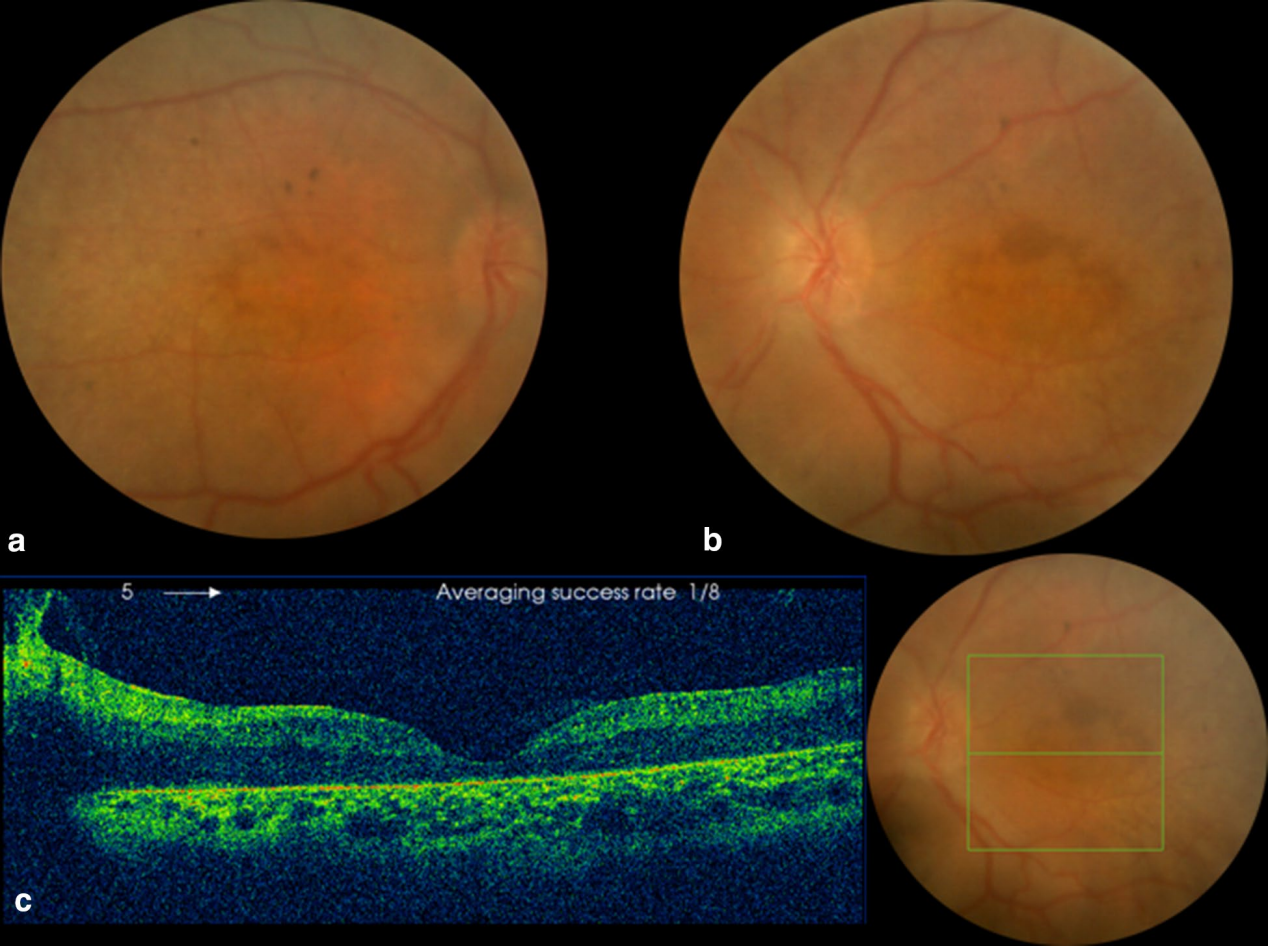
Fundus Photo (*OU*) and OCT (*OS*) of the 12 year old male sibling. **a**, **b** Fundus photos showing foveal atrophy, nummular pigmentations and moderate vascular attenuation. **c** OCT showing decreased foveal thickness

**Fig. 4:**
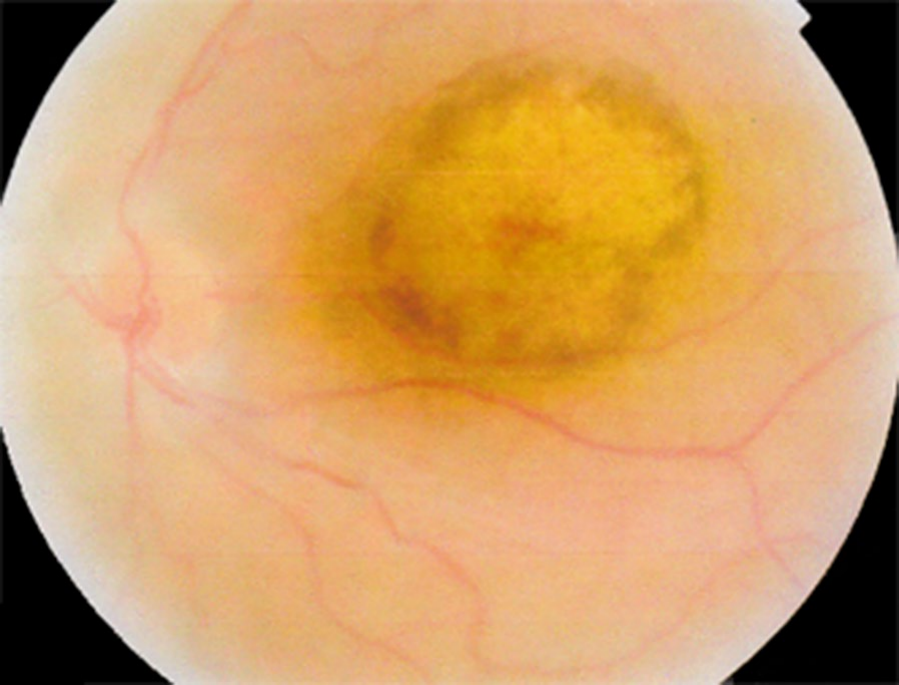
Fundus photo of the 2 year old male sibling showing foveal atrophy

ERG revealed severely reduced scotopic and photopic responses; suggestive of a severe form of rod-cone dystrophy consistent with LCA. Ocular coherence tomography (OCT) revealed serous retinal detachment involving the fovea in left eye.

Examination of family members revealed two younger male siblings (12 years old, and 2 years old) having profound visual loss. The clinical presentation of the two siblings is presented in Table 1, and it was suggestive of LCA, one more sibling (8 years old male) and the two parents revealed no ocular abnormalities.

**Table 1:** Clinical presentation of the two younger siblings

No.	Age	Sex	BCVA OD/OS	Cyclorefration OD/OS	RAPD	AxL (mm) OD/OS	Fundus findings	ERG findings	OCT findings
1	12 years	Male	CF/0.16	S + 8.00 C −1.00×10° / S + 8.00 C −4.00×175°	No	18.30/18.35	Foveal atrophy, nummular pigmentations, moderate vascular attenuation (Fig. 3a, b)	Cone-rod dystrophy, LCA	Decreased foveal thickness (Fig. 3c)
2	2 years	Male	N/A	S + 10.00 C −2.25×125°/ S + 10.00 C−2.50×25°	No	18.40/18.35	Foveal atrophy (Fig. 4)	Leber congenital amaurosis, cone-rode dystrophy	N/A

*BCVA* best corrected visual acuity, *RAPD* relative afferent pupillary defect, *AxL* axial length, *ERG* electroretiography, *OCT* ocular coherence tomography, *CF* counting fingers, *N/A* not available

*CRB1* mutation was suspected to be responsible for the pathologic presentations of the patients, and gene sequencing was performed searching for mutations in the entire coding sequence of the *CRB1* gene using fluorescent sequencing, which revealed that the three siblings share the same variant *c.2555T* >*C (p.I852T)* in the homozygous state. Accordingly, diagnosis of LCA8 was confirmed in the three siblings.

As there are no definitive guidelines for the management of coat’s like vasculopathy associated with retinal dystrophies, decision was made to perform pan-retinal photocoagulation (PRP) over the inferior detached retina in the left eye with close follow up, and only close follow up in the right eye as the parents refused any type of management in the better seeing eye. The retinal detachment remained stable over the following 6 weeks. On the eighth week post laser the patient complained of sudden decrease of visual acuity in her better right untreated eye to perception of light (PL) with no right projection, and to no perception of light (NPL) in the left previously treated eye. The decision to perform pars plana vitrectomy (PPV) for the right eye was made, advantages and disadvantages of surgical treatment were explained to the patient and parents and informed consent was taken. Standard PPV with extensive intraoperative PRP was done with the use of silicone oil tamponade at the end of surgery. No surgical treatment was done for the left eye as the patient refused surgery for this eye.

Over 6 months follow up period, the retina of the right eye remained attached with no improvement in visual acuity (perception of light with no light projection), while the left eye continued to have subtotal retinal detachment, which appeared to increase in height and finally developed neovascular glaucoma.

## Discussion

LCA is the earliest and most severe form of all inherited retinal dystrophies, characterized by blindness or severe visual impairment from birth [2]. LCA has a variable expression which may reflect, at least in part, its high genetic heterogeneity, disease-associated mutations have been reported in at least seventeen genes including: *GUCY2D, CRB1, RPE65, RPGRIP1, AIPL1, TULP1 and CRX* [6]. Crumbs homologue 1 (*CRB1*) is homologous to the Drosophila Crumbs protein and Drosophila Crumbs is essential for establishing and maintaining apico-basal polarity in embryonic epithelia derived from the ectoderm. In particular, Crumbs is required for the biogenesis of the zonula adherens, a belt-like structure encircling the apex of epithelial cells [2].

*CRB1* mutations are the second most common gene mutations found among LCA patients, accounting for 7.4–10 % of all LCA patients [7, 8], and mutations in the *CRB1* gene are associated with variable phenotypes of severe retinal dystrophies, ranging from LCA to RP.

The association of coat’s like vasculopathy and RP is extensively studied, a complication that is estimated to be found in 1.2–3.6 % of all RP patients [5]. *CRB1* mutations were found in about 55 % of these patients [6], suggesting that *CRB1* mutations should be considered an important risk factor for the coats-like reaction in RP patients, although its development may require additional genetic or environmental factors [6]. On the other hand, the presence of coat’s like vasculopathy is highly suggestive of a *CRB1* mutation, and *CRB1* sequencing may be warranted in these patients.

Our patient presented with bilateral asymmetric coat’s like picture, high hyperopia, posterior microphthalmos and severely reduced scotopic and photopic ERG responses which was suggestive of a severe retinal dystrophy. This presentation suggested that *CRB1* may be the responsible gene. *CRB1* gene sequencing for the patient and her affected siblings revealed a homozygote state of the variant c.2555T >C (p.I852T).

This same mutation was mentioned in the literature by Francesca Simonelli et al. [8] who thoroughly studied 95 patients with LCA, and found *CRB1* mutations in 7.4 % of them, with the same p.I852T mutation (of our patients) in two patients of Italian ancestry, who were 40 and 45 years old and had severe loss of vision: perception of light in the first and hand movement in the second. In addition both patients had cycloplegic refraction of about +4.00 diopters and ERG responses compatible with Rod-Cone dystrophy. The two patients in Simonelli study were described to have “retinitis pigmentosa fundus findings”, whereas our patient presented with the coat’s like vasculopathy and nummular pigmentations, and the two siblings presented with macular atrophy and nummular pigmentations.

Searching the literature for suggested management of patients having coat’s like disease and RP revealed multiple management plans including: vitrectomy and endolaser [9], cryotherapy, scleral buckling with subretinal fluid drainage and laser photocoagulation of the telangiectatic and neovascular lesions [10] and intravitreal dexamethasone implant [11].

Our patient was managed initially with PRP over the detached retina in the worse (left) eye as the detachment was not involving the macula. Later as the case progressed to total retinal detachment in both eyes with the right eye being the better one, a PPV, endolaser and silicone oil tamponade was done for the right eye trying to reattach the retina with resultant anatomical stabilization defined by maintaining attached retina and prevention of neovascular glaucoma in this eye, which was encountered in the other non- operated eye. Retinal reattachment was not accompanied with any improvement in visual function or restoring the previous vision.

We suggest PPV with extensive endolaser to be considered early in the course of such patients as the severe subretinal exudates and the extensive exudative detachment makes the PRP most likely not efficient.

## Conclusion

*CRB1* gene mutation is a very well known risk factor for the development of coat’s like vasculopathy in RP patients, and our case presents the co-existence of the coat’s like reaction and LCA in a patient with *CRB1* mutation, it is not obvious whether this mutation is responsible for the coat’s reaction as it was also described in other patients without causing the coat’s exudate.

LCA has a very guarded prognosis, but it can be even worse when a complication such as coat’s like vasculopathy accompanies the case. We suggest close follow up for all LCA patients who have *CRB1* mutations, especially the *c.2555T* >*C (p.I852T)* homozygote variant searching for any vascular abnormality and managing it as soon as it is diagnosed with PPV and endolaser in an attempt to halt the complete loss of the already severely affected visual function, preserve the eye and prevent neovascular glaucoma and painful blind eye.

## Consent

Written informed consents were obtained from the patient and from the father of the siblings for publication of this case report and any accompanying images.

## Abbreviations

LCA: leber congenital amaurosis
ERG: electroretinogram
RP: retinitis pigmentosa
FA: fluorescein angiography
OCT: ocular coherence tomography
PRP: panretinal photocoagulation
PL: perception of light
NPL: no perception of light
PPV: pars plana vitrectomy

## References

[1] Leber T (1869). Ueber retinitis pigmentosa und angeborene Amaurose. Graefes Arch Clin Exp Ophthalmol.

[2] Cremers FP, van den Hurk JA, den Hollander AI (2002). Molecular genetics of Leber congenital amaurosis. Hum Mol Genet.

[3] De Laey JJ (1991). Leber’s congenital amaurosis. Bull Soc Belge Ophtalmol.

[4] Bujakowska Kinga, Audo Isabelle, Mohand-Saïd Saddek, Lancelot Marie-Elise, Antonio Aline, Germain Aurore, Léveillard Thierry, Letexier Mélanie, Saraiva Jean-Paul, Lonjou Christine, Carpentier Wassila, Sahel José-Alain, Bhattacharya Shomi S, Zeitz Christina (2012). CRB1 mutations in inherited retinal dystrophies. Hum Mutat.

[5] Khan JA, Ide CH, Strickland MP (1988). Coats’-type retinitis pigmentosa. Surv Ophthalmol.

[6] den Hollander AI, Heckenlively JR, van den Born LI, de Kok YJ, van der Velde-Visser SD, Kellner U, Jurklies B, van Schooneveld MJ, Blankenagel A, Rohrschneider K, Wissinger B, Cruysberg JR, Deutman AF, Brunner HG, Apfelstedt-Sylla E, Hoyng CB, Cremers FP (2001). Leber congenital amaurosis and retinitis pigmentosa with coats-like exudative vasculopathy are associated with mutations in the crumbs homologue 1 (CRB1) gene. Am J Hum Genet.

[7] Hanein S, Perrault I, Gerber S, Tanguy G, Barbet F, Ducroq D, Calvas P, Dollfus H, Hamel C, Lopponen T, Munier F, Santos L, Shalev S, Zafeiriou D, Dufier JL, Munnich A, Rozet JM, Kaplan J (2004). Leber congenital amaurosis: comprehensive survey of the genetic heterogeneity, refinement of the clinical definition, and genotype-phenotype correlations as a strategy for molecular diagnosis. Hum Mutat.

[8] Simonelli F, Ziviello C, Testa F, Rossi S, Fazzi E, Bianchi PE, Fossarello M, Signorini S, Bertone C, Galantuomo S, Brancati F, Valente EM, Ciccodicola A, Rinaldi E, Auricchio A, Banfi S (2007). Clinical and molecular genetics of Leber’s congenital amaurosis: a multicenter study of Italian patients. Invest Ophthalmol Vis Sci.

[9] Bansal S, Saha N, Woon WH. The management of “coats’ response” in a patient with x-linked retinitis pigmentosa-a case report ISRN Surg 2011, 2011:970361. 10.5402/2011/970361 PMC3200260 22084788

[10] Kan Emrah, Yilmaz Turgut, Aydemir Orhan, Güler Mete, Kurt Jülide (2007). Coats-like retinitis pigmentosa: reports of three cases. Clin Ophthalmol.

[11] Patil L, Lotery AJ (2014). Coat’s-like exudation in rhodopsin retinitis pigmentosa: successful treatment with an intravitreal dexamethasone implant. Eye (Lond).

